# Genomic background selection to reduce the mutation load after random mutagenesis

**DOI:** 10.1038/s41598-021-98934-5

**Published:** 2021-09-30

**Authors:** Nirosha L. Karunarathna, Dilan S. R. Patiranage, Hans-Joachim Harloff, Niharika Sashidhar, Christian Jung

**Affiliations:** 1 Plant Breeding Institute, Christian-Albrechts-University of Kiel, Olshausenstr. 40, 24098 Kiel, Germany; 2 Plant Breeding Institute, Christian-Albrechts-University of Kiel, Olshausenstrasse 40, 24118 Kiel, Germany; 3 Present Address: KWS LOCHOW GMBH, Zuchtstation Wetze, Wetze 3, 37154 Northeim, Germany; 4 Present Address: Max Planck Institute for Plant Breeding Research, Carl-von-Linne-Weg 10, 50829 Cologne, Germany

**Keywords:** Genomic analysis, Agricultural genetics, Genetics, Genetic markers, Plant breeding, Plant genetics

## Abstract

Random mutagenesis is a standard procedure to increase allelic variation in a crop species, especially in countries where the use of genetically modified crops is limited due to legal constraints. The chemical mutagen EMS is used in many species to induce random mutations throughout the genome with high mutation density. The major drawback for functional analysis is a high background mutation load in a single plant that must be eliminated by subsequent backcrossing, a time and resource-intensive activity. Here, we demonstrate that genomic background selection combined with marker-assisted selection is an efficient way to select individuals with reduced background mutations within a short period. We identified BC_1_ plants with a significantly higher share of the recurrent parent genome, thus saving one backcross generation. Furthermore, spring rapeseed as the recurrent parent in a backcrossing program could accelerate breeding by reducing the generation cycle. Our study depicts the potential for reducing the background mutation load while accelerating the generation cycle in EMS-induced winter oilseed rape populations by integrating genomic background selection.

## Introduction

Oilseed rape (*Brassica napus*) is an important oil crop grown worldwide with a broad adaptation to different climates and daylength regimes. Winter oilseed rape is mainly grown in Europe. It only flowers after a period of cold exposure. In contrast, spring oilseed rape, grown primarily in Canada and Australia, does not need vernalization for flowering. Semi-winter oilseed rape is widely cultivated in China and requires moderate vernalization. The demand for oilseed rape has increased over the years, as oilseed rape is not only used for the production of edible oil but also as a protein source for feed, biofuel, and industrial raw material^1^. The generation cycle of winter rapeseed is much longer because it needs vernalization for flowering. Under field conditions, winter rapeseed requires one year to complete its life cycle, while in the greenhouse, six to seven months are needed for seed production. In contrast, early spring accessions need 3–4 months to produce seeds in the greenhouse yielding three to four generations per year^2^. Rapid generation cycling *B. napus* has been proposed, producing up to six generations per year (55 days generation cycle) under high light conditions in the greenhouse at 24 °C^3^.

Oilseed rape is a young crop with a short history of cultivation and breeding^4^. Genetic variation within this species is considerably small^5^. Mutagenesis is a powerful tool for creating new genetic variation. CRISPR-Cas mediated mutagenesis has been successfully applied to develop new allelic variation in oilseed rape^6–8^. However, its practical application is hampered by legal constraints in the European Union and other countries^9^. Therefore, chemical or irradiation-induced random mutagenesis is an important alternative. Numerous mutants have been produced with improved agronomical characters such as shatter resistance, flowering time, seed weight, and yield components^10–13^. However, the usage of mutants in breeding programs is limited due to their high mutation load. In our EMS rapeseed population, each plant has ~ 46,000 mutations^6^. While they can be grown in the greenhouse to study the effect of a mutation in a target gene, their application for field studies is limited. Plant development and flowering time are delayed, and they are suffering from low vigor and poor abiotic stress tolerance. In our field studies, they did not even survive the winter. The mutation load is even more increased after crossing different mutants to produce double mutants. This is the only way to get a new phenotype in polyploids because single mutants do not show a phenotypic effect^6^. Therefore, the EMS donor genome must be replaced by an elite genome, which requires at least 4–6 generations of backcrossing^14^.

To accelerate breeding, breeders use different methods like single seed descent (SSD) in pedigree breeding and doubled haploid (DH) technology^15^. A recent study claimed to accelerate crop research and breeding by "speed breeding"^2^. Among other species, they grew canola (*B. napus*) under prolonged photoperiods with a day length of 22 h in a temperature-controlled glasshouse fitted with high-pressure sodium lamps. Under these controlled-environmental conditions, they reduced generation cycles for spring canola to 73 $\pm$ 9 days^2^. Thus, four generations per year could be achieved.

Recurrent backcrossing is commonly used to transfer genes responsible for favorable agronomic traits from a donor line to the recurrent parent^14^. Typically, the recurrent parent is an elite line with low relatedness to the donor parent. Marker-assisted selection enables efficient detection of the target gene. In contrast, genomic background selection retains the original characters of the recurrent parent, thus reducing the number of generation cycles needed to obtain an improved elite line^16,17^. High throughput marker genotyping technologies are used in oilseed rape breeding and research^18,19^. *B. napus* Illumina Infinium arrays ranging from 6 to 60 K^20–23^ have been applied in germplasm genotyping^24^, genome-wide association studies^25–28^, and QTL mapping^29,30^.

Here, we propose genomic background selection in early backcross generations. Offspring from EMS treated rapeseed plants were backcrossed with an elite line. Different backcross populations were genotyped with a 15 K Illumina Infinium array. Plants with a high share of the backcross parent genome were identified in the first backcross generation. This study demonstrates that plants with substantially lower mutation load can be selected from the first backcross generation. The application for breeding with EMS mutagenized plants is discussed.

## Results

### Marker-assisted foreground selection of mutant plants in segregating populations

We produced segregating populations with a varying share of the mutagenized donor genomes either by backcrossing or crossing two F_1_ hybrids carrying different mutant alleles. We selected six different EMS-induced nonsense mutants for backcrossing with Peace, two *BnSFAR4-a* mutants (*Bna.SFAR4.A06a* and *Bna.SFAR4.C03a*)^6^, two *Bn2-PGK2* mutants (*Bna.2-PGK2.A02* and *Bna.2-PGK2.A10*)^31^, and two *BnMRP5* mutants (*Bna.MRP5.A10* and *Bna.MRP5.C05*)^32^ (Supplementary Table 1). In the case of *BnSFAR4-a*, to produce BC_1_ populations, we backcrossed F_1_ hybrids (M_3_ × Express 617 mutant) with Peace, while for *BnMRP5* and *Bn2-PGK2*, we backcrossed M_3_ mutants with Peace (Supplementary Fig. 1). We also generated F_1_ × F_1_ double mutants for *BnMRP5* and *Bn2-PGK2* by combining F_1_ hybrids after crossing the M_3_ mutant with Peace. In total, we selected seven families for marker-assisted backcross selection. For easier understanding, we used a short letter code A through G (Table 1).

**Table 1:** Recurrent parental genome contribution in seven BC_1_ and F_1_ oilseed rape generations.

Gene name	Population (seed code)	Generation	Number of plants genotyped	Recurrent parent genome share		Plants with higher Peace genome share
				Per plant (%)	Average (%)	
*Bna.SFAR4.A06a*	A (180901)	BC_1_	85	68.3–83.7	75.4	50 (58.8%)
*Bna.SFAR4.C03a*	B (180902)	BC_1_	99	65.7–84.5	74.8	48 (48.5%)
*Bna.2-PGK2.A10*	C (183295)	BC_1_	33	69.6–85.7	79.0	26 (78.8%)
*Bna.MRP5.A10*	D (180858)	BC_1_	20	67.8–81.6	75.5	12 (60.0%)
*Bna.MRP5.C05*	E (180837)	BC_1_	20	71.5–80.6	75.8	11 (55.0%)
*Bn2-PGK2*	F (183297)	F_1_ × F_1_	94	42.5–65.3	54.1	75 (79.8%)
*BnMRP5*	G (180859)	F_1_ × F_1_	60	45.2–65.4	54.9	50 (83.3%)

Genome share was calculated as % of the Peace genome. In the last column, plants with a higher Peace genome share were defined as plants with more than the expected average.

We questioned whether F_1_ plants from a single cross with Peace would flower without vernalization. Therefore, *BnSFAR4-a* single mutant F_1_ and BC_1_ plants were grown in the greenhouse (16 h light/ 8 h dark, 23–24 °C) without vernalization. Four months after sowing, none of the F_1_ plants had entered the generative phase (data not shown).

We selected BC_1_ and F_1_ × F_1_ plants carrying the mutant alleles using allele-specific markers and Sanger sequencing (foreground selection) (Supplementary Table 2). Expected 1:1 and 1:1:1:1 segregation ratios for the mutant alleles were confirmed for BC_1_ (populations A, C, D, and E) and F_1_ × F_1_ (populations F and G), respectively, except for population B. However, the number of heterozygous mutants in this population was sufficient for genomic background selection. In summary, we identified 256 heterozygous plants in the BC_1_ populations and 154 heterozygous double mutants in the F_1_ × F_1_ populations.

### Population structure analysis

We genotyped 256 BC_1_ and 154 F_1_ × F_1_ plants carrying the mutant alleles along with M_3_/F_1_ mutant parents and the non-mutagenized Express 617 and recurrent Peace parents by using the SNP array resulting in 13,416 functional SNPs (Table 2). From populations C through G, F_1_ (M_3_ × Peace) hybrids were taken as parental controls (Supplementary Fig. 1). Monomorphic markers are not informative as they cannot distinguish between parental genotypes. Therefore, we filtered SNPs for minor allele frequencies of 0.001 to exclude both genotyping artifacts and monomorphic markers, which resulted in 7,686 informative SNPs. Of these, 6,887 were polymorphic between Peace and Express 617 (Table 2).

**Table 2:** Results from genotyping BC_1_ and F_1_ × F_1_ populations with the 15 K Illumina Infinium SNP array.

	Number of SNPs
Markers on the SNP array	13,714
Functional markers for BC_1_ and F_1_ × F_1_ genotyping	13,416
Markers after filtering	7686

Functional markers are the markers without missing SNP data. SNP information filtering was done for a minor allele frequency of 0.001.

**Figure 1:**
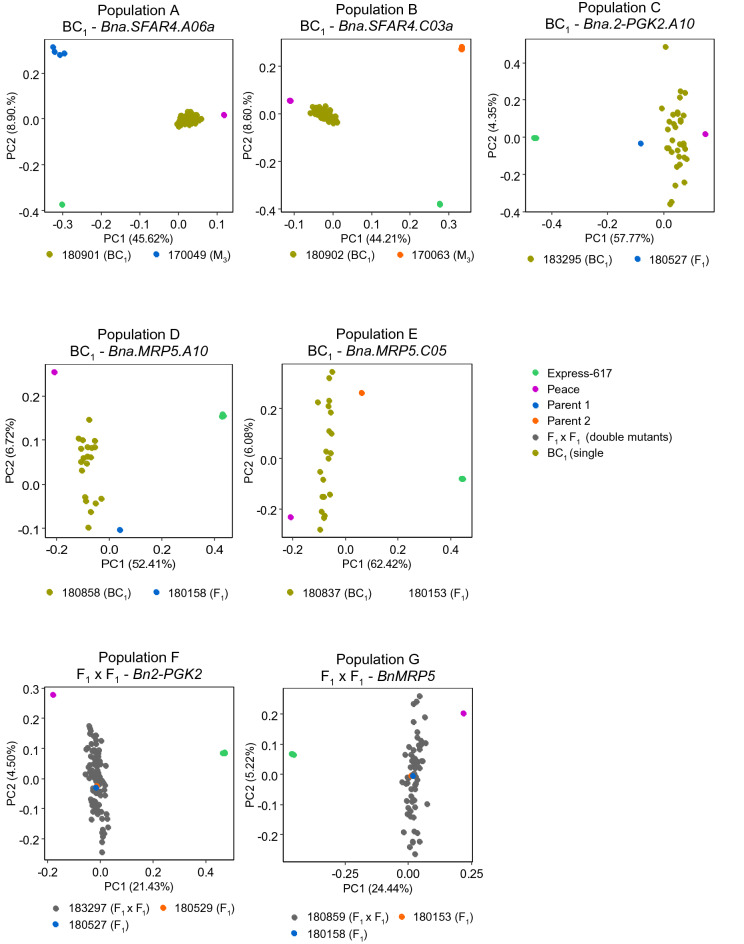
Principal component (PC) analysis with five BC_1_ single mutant and two F_1_ × F_1_ double mutant oilseed rape populations. PCA was performed for each population separately, and PC1 and PC2 were displayed using the ggplot2 package in R. Seed codes of populations and respective parents are given at the bottom of each plot. Size of each population as follows, population A = 85, population B = 99, population C = 33, population D = 20, population E = 20, population F = 94, and population G = 60. M_3_ mutants were used as the parents for populations A and B, while F_1_ (M_3_ × Peace) hybrids were taken as parental controls for populations C to G.

Then, we analyzed the structure of seven populations. A principal component (PC) analysis was carried out separately for each population. The first two PCs (PC1 and PC2) explained 25.93% to 68.50% of the population's total genetic variation (Fig. 1). We expected to observe four and three clusters in the BC_1_ and F_1_ × F_1_ populations, respectively. Based on the top two PCs, we detected in all BC_1_ single mutant populations four main clusters, i.e., Express 617, Peace, parents, and BC_1_ (Fig. 1). F_1_ × F_1_ double mutant populations displayed three main clusters, Express 617, Peace, and F_1_ double mutants. In these populations, parents were clustered together with F_1_ mutants. As expected, backcrossed plants and the recurrent genotype Peace were less distant from each other, indicating that backcrossed plants carried a higher Peace background percentage. In populations A and B, Express 617 and M_3_ parents were clustered along with the second principal component, reflecting low genetic variation between these genotypes (Fig. 1).

### Genome composition in backcross generations

**Figure 2:**
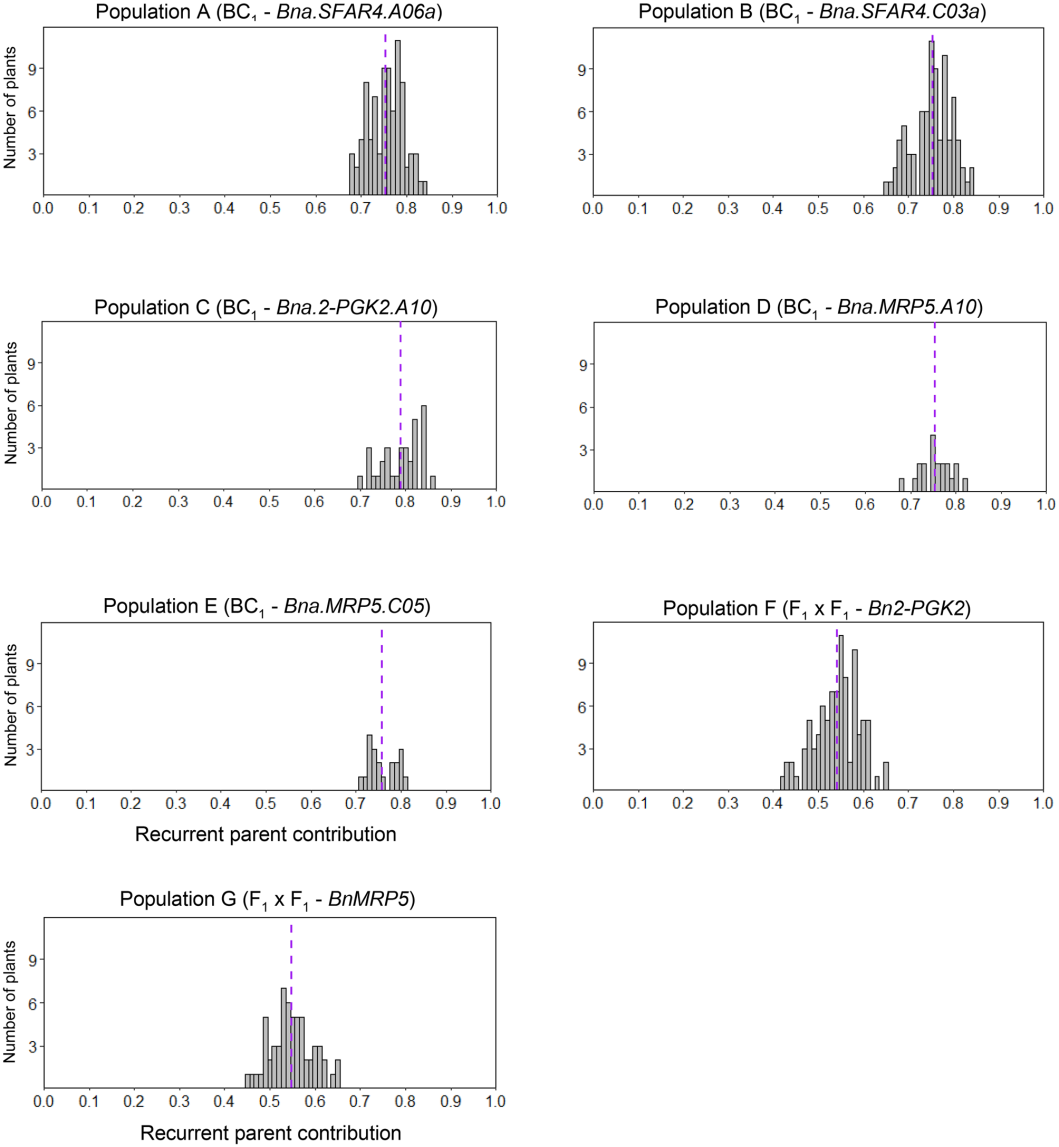
Proportion of the recurrent parent in five BC_1_ single mutant and two F_1_ × F_1_ double mutant populations. The purple lines indicate the average of the recurrent parent's genome share.

We aimed to select the plants carrying the highest Peace genomic background. Therefore, we calculated the proportion of accumulated recurrent and donor parents' genomes for each plant. In a BC_1_ generation, the expected share of the recurrent parent's genome is 75%. In the respective populations (A, B, C, D, and E), the proportion of the backcross parent genome ranged between 65.7% and 85.7% (Fig. 2, Table 1 and Supplementary data 1). The average share in four populations (A, B, D, and E) ranged from 74.8 to 75.8%, which is very close to the expected value. A slightly higher value was calculated for population C (79.0%). In the double-hybrid populations, the expected share of the Peace genome is 50%. The values calculated for individual plants of populations F and G ranged from 42.5% to 65.4% (Fig. 2 and Table 1). The average share of the recurrent parent genome in populations F and G was 54.1% and 54.9%, which is higher than the expected value (50%).

This study's primary aim was to select plants with a higher than average share of the backcross parent (Peace) genome. Across all BC_1_ populations, the proportion of plants meeting these expectations ranged between 48.5% and 78.8% (Table 1). In the F_1_ × F_1_ double mutant populations F and G, the frequency of plants with a Peace genome share above 50% was 79.8% and 83.3%, respectively (Table 1). Based on these results, individual plants with substantially higher backcross genome share could be selected from the first segregating generations.

## Discussion

Repeated backcross breeding is a common practice that many breeders use to incorporate new alleles into elite plant material. However, conventional backcrossing is a time-consuming procedure, mainly in biennial species with long generation cycles. This study demonstrates the potential of genomic background selection for introducing EMS-induced alleles into oilseed rape breeding lines with a fast reduction of background mutation load.

A high mutation load is a major problem arising with EMS mutagenized plants^6,33^. Hence, background mutations should be eliminated by subsequent backcrossing with elite germplasm. The winter oilseed rape in this study has a strong vernalization requirement, which takes a minimum of six months to complete one generation under greenhouse conditions. Repeated backcrossing is required to produce a mutant line with a low mutation load. Here, we investigated two parameters to shorten the period for producing an improved elite line with a substantially reduced mutation load. First, we used a spring-type line as a recurrent parent, saving three months per generation compared to a cross with a winter-type. Applying the single seed descent (SSD) method under highly controlled growth conditions has reduced generation time in wheat and barley^2^. Harvesting and germination of the immature seeds, thereby shortening the generation cycle, have been proven for wheat, pigeon pea, and faba bean^2,34,35^. Hence, there is a potential for further reducing the generation cycle, at least by three to four weeks in our oilseed rape populations, by combining SSD with immature seed harvesting and germination under greenhouse conditions. This approach, however, is only applicable for research projects where the effect of mutant alleles is studied among a non-mutated genome background. In the European winter breeding program, backcrossing with spring type is avoided because of their non-adapted flowering time.

Then, we applied marker-assisted selection to save repeated cycles of backcrossing, which included two steps, foreground selection for the mutant allele and genomic background selection for the recurrent parent genome. We chose the 15 K Illumina Infinium SNP array for genotyping for two reasons. First, the information content is high because thousands of loci can be genotyped within a short time. Second, co-dominance enables identifying heterozygous and homozygous loci, which is essential in BC_1_ populations. The recovery rate of the recipient genome by marker-assisted backcrossing relies on the number of backcross generations, marker density, and population size^17^. In general, higher recovery of the recipient genome can be obtained in early backcross generations using high marker densities and large populations^36^. The marker density in this study was around 6.09 markers/Mbp, which is far enough for background selection. In an earlier study, it has been shown that even a lesser number of markers and plants are sufficient for high response to selection^37^. Low-cost multiplex marker systems such as AFLPs could offer an alternative; however, the workload is much higher than for SNP arrays.

On average, in the BC_1_ generation, a 75% recurrent parent genome is expected with one target gene introgression^38^. Our results met the expectations based on common genetic knowledge. Similar data were reported for rice, wheat, and maize^39–41^. As a side effect, our results demonstrate the accurateness of hand crosses because we found no self-pollination events.

It has been shown that marker-assisted background selection accelerates the recovery of the recurrent parent genome^38,42,43^. We could identify a BC_1_ plant with a recurrent genome share of 85.7%, the approximate average recipient genome share in a BC_2_ generation. Thus, one generation of backcrossing could be saved, leading to higher genetic gain. In polyploids, single mutations rarely have a phenotypic effect due to gene redundancy^6,31,44^. If gene families are targeted, a minimum of two knockout alleles must be combined in one genome, as has recently been demonstrated for *BnSFAR4* and *Bn2-PGK2* gene families in rapeseed^6,31^. Although CRISPR-Cas mediated mutagenesis turned out to be superior, random mutagenesis like EMS is the only choice for European breeders to work with novel alleles due to legal constraints^9^. Therefore, the F_1_ × F_1_ double mutant hybrids of our study are of high practical relevance. We could select individual plants with 65% of the recovered recurrent parent's genome from these populations, far exceeding the theoretical average (50%). The reason could be that these populations are derived from two meiotic generations resulting in more recombination events during the meiosis.

In conclusion, we suggest using double hybrid populations for marker-assisted background selection to reduce the mutation load after random mutagenesis. This study was a proof of principle experiment with a considerably low number of plants. We expect that plants with higher recurrent genome share will be identified in practical breeding programs working with much larger populations.

## Materials and methods

### Plant materials and crossing scheme

We used previously identified EMS-induced single mutants of winter oilseed rape (Supplementary Table 1) as the primary materials to perform different crosses. Seed fatty acid reducer (*SFAR*) genes encode lipases active during seed development. Gene knockout resulted in elevated seed oil contents^6^. The *multi-drug resistant protein 5* (*MRP5)* encodes a phytic acid transporter protein whereas the *2-phosphoglyceric acid kinase gene* (*2-PGK*) encodes an enzyme that is part of the phytic acid biosynthesis pathway. Knockout of both genes resulted in drastically reduced phytic acid seed contents^31,32^. We crossed *BnSFAR4* M_3_ mutants with Express 617, which is the EMS donor genotype. The resulting F_1_ plants were backcrossed twice with the Canadian spring variety ‘Peace’ to produce BC_1_ populations (Supplementary Fig. 1). We used ‘Peace’ for backcrossing because it is flowering very early in contrast to adapted German varieties which need a long time for vernalization. The mutant’s agronomic value could only be assessed in backcross generations. *BnMRP5* and *Bn2-PGK2* M_3_ mutants were first crossed with Peace to produce F_1_ plants. The F_1_ plants were crossed with each other within a subfamily to produce double mutants (F_1_ × F_1_) or backcrossed with Peace to produce BC_1_ single mutants (Supplementary Fig. 1). Seeds were sown in 3 × 3 cm 35-multi-well palettes in the greenhouse, and plants were grown in 9 × 9 cm pots for seed production. Plants were grown under greenhouse conditions (16 h light/8 h dark, 23–24 °C). For vernalization, plants were transferred to a cold chamber (16 h light/ 8 h dark, 4 °C) for eight weeks and then returned to the greenhouse. Plastic selfing bags were mounted after bolting to control pollination.

### DNA isolation and selection of mutant plants

Leaf genomic DNA was extracted from greenhouse-grown plants using the cetyltrimethylammonium bromide (CTAB) method^45^. We used allele-specific markers to select mutant genotypes for genotyping BC_1_ and F_1_ × F_1_ plants (Supplementary Table 2). PCR was performed under the following cycling conditions: 94 °C for 3 min, 40 cycles of 94 °C for 30 s, 60–66 °C for 30 s, and 72 °C for 1 min, followed by 72 °C for 5 min final elongation, and PCR products were separated on agarose gels. PCR products carrying the mutant alleles were Sanger sequenced (Institute of Clinical Molecular Biology, Kiel) to confirm the mutations.

### Genotyping with the 15 K Illumina Infinium SNP array

The genomic DNA of mutant plants was normalized to a concentration of 50–200 ng/µl by using a NanoDrop2000 spectrophotometer (ThermoFisher Scientific, Waltham, United States). From the normalized DNA of BC_1_ and F_1_ × F_1_ plants, 20 µl aliquots were sent for genotyping (TraitGenetics GmbH, Gatersleben, Germany) using the *Brassica* 15 K Illumina Infinium SNP array (Illumina, San Diego, CA).

Genotyping data were displayed in an MS Excel file format. First, we converted the Excel files to the Hapmap (Haplotype Map) format applying customized R scripts. Using TASSEL (Trait Analysis by aSSociation, Evolution and Linkage)^46^, the Hapmap file was converted into the VCF (Variant Call Format) format^47^. We filtered SNPs for minor allele frequencies lower than 0.001 using the VCFtools^47^. With this filtering, we attained the VCF file with high-quality SNPs.

We performed Principal Component (PC) analysis using the SNPRelate package^48^ in R 3.6.1^49^. First, two PCs were plotted using the ggplot2 package in R (Wickham H (2016)^50^. Using the TASSEL software, we converted all alleles into the ABH file format. The Peace and Express 617 alleles are written as '*A*' and '*B*', respectively, and '*H'* represents heterozygosity. Then, genotypes were exported to a CSV (comma-separated values) file. The resulting genotype file was used to calculate the share of the Peace and Express 617 genome (Supplementary data 1), following the formula.

$$\% \,{\text{of}}\,{\text{parent}}\,{\text{genome}} = \frac{{\left( {{\text{homozygous}}\,{\text{allele}} \times 2} \right) + \left( {{\text{heterozygous}}\,{\text{allele}} \times 1} \right) }}{{{\text{total}}\,{\text{number}}\,{\text{of}}\,{\text{alleles}}}} \times 100$$

### Statement for the plant materials

The authors confirmed that the collection of plant material or the collection of seeds in the study complies with relevant institutional, national, and international guidelines and legislation.

## Supplementary Information

Supplementary Information.

## Data Availability

The authors declare that data supporting the finding of this study are available from this manuscript and its supplementary information files. Extra data, information, and plant materials used/produced in this study are available from the corresponding authors upon request.
