# Supplementary material for: Genomic background selection to reduce the mutation load after random mutagenesis
Source: Sci Rep. 2021 Sep 30;11:19404. doi: 10.1038/s41598-021-98934-5 (PMC8484577; doi:10.1038/s41598-021-98934-5)
Supplement: Supplementary file 1 — Supplementary Information. [file 41598_2021_98934_MOESM1_ESM.pdf]

Supplementary Table 1: M_3 mutants used for crosses

M_3 seed code	*B. napus* paralog	Amino acid substitution	Allele name[1]	Amino acid Changes	Reference
170049	*Bna.SFAR4.A06a*	Q93*	C_1	Glutamine to stop codon mutation	[1]
170063	*Bna.SFAR4.C03a*	Q93*	D_1	Glutamine to stop codon mutation	[1]
170461	*Bna.2PGK2.A02*	Q269*	H_1	Glutamine to stop codon mutation	[2]
170492	*Bna.2PGK2.A10*	Q283*	I_1	Glutamine to stop codon mutation	[2]
170482	*Bna.MRP5.A10*	W359*	J_1	Tryptophan to stop codon mutation	[3]
170481	*Bna.MRP5.C05*	Q210*	K_1	Glutamine to stop codon mutation	[3]

Supplementary Table 2: Allele-specific primers used for genotyping. Red color nucleotides designate the mismatches introduced, according to[4], for increasing primer specificity.

***B. napus* paralog**	**Primer name**	**Primer sequence**	**Orientation**	**Primer combination**	**Primer specificity**
Bna.SFAR4.A06a	NK023	CATCTTCTGCAGCCAGCGACAAT	reverse		paralog specific
	NK134	CGACTCACCATTGACTTCGTGGAGT	forward	NK134+NK023	mutant specific
	NK142	CGACTCACCATTGACTTCGTGGAGC	forward	NK142+NK023	wild type specific
Bna.SFAR4.C03a	NK021	CATCTTCTGCAGCCAGCGACTTC	reverse		paralog specific
	NK136	GCGACTCACCATTGACTTCGTGGAGT	forward	NK136+NK021	mutant specific
	NK144	GCGACTCACCATTGACTTCGTGGAGC	forward	NK144+NK021	wild type specific
Bna.2PGK2.A02	NS_P248	TCTCGCTGAATATAAGTACGTCTGC	forward		paralog specific
	2PGK.A02_M	GAGATTCTCAATCACCATCTCACTCTA	reverse	NS_P248+2PGK.A02_M	mutant specific
	NS_P174	AGAGGCCAAGTTCTTGAGAAAGTT	reverse		paralog specific
	2PGK.A02_W	GCTGTGGAAGGATTCAAAGGTC	forward	2PGK.A02_W +NS_P174	wild type specific
Bna.2PGK2.A10	NS_P172	CGGCCAAGTTCTTGCAAAAGTC	reverse		paralog specific
	2PGK.A10_M	ACTCCTGTATTGTTAAGCCACATGT	forward	2PGK.A10_M +NS_P172	mutant specific
	2PGK.A10_W	CCTGTATTGTTAAGCCACATGC	forward	2PGKA10_W +NS_P172	wild type specific
Bna.MRP5.A10	NS_P252	ACGTGCGATTCTCAAGTCCCTT	forward		paralog specific
	MRP5.A10_M	TCAAGATATCAACCCCCATGAAT	reverse	NS_P252+MRP5.A10_M	mutant specific
	MRP5.A10_W	CAAGATATCAACCCCCATGAAC	reverse	NS_P252+MRP5.A10_W	wild type specific
Bna.MRP5.C05	NS_P313	GACTTGGCTCTATCCCTCAGC	reverse		paralog specific
	MRP5.C05_M	GAAATTCGTCTAACCTCTTCTGTTCATT	forward	MRP5.C05_M+NS_P313	mutant specific
	MRP5.C05_W	GAAATTCGTCTAACCTCTTCTGTTCATC	forward	MRP5.C05_W+ NS_P313	wild type specific

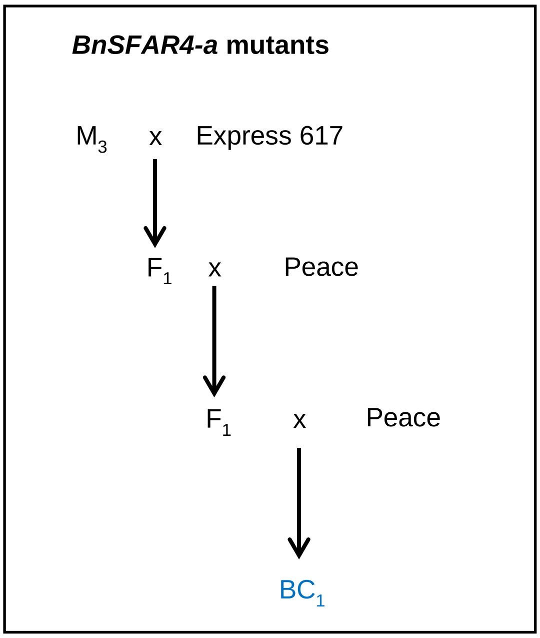

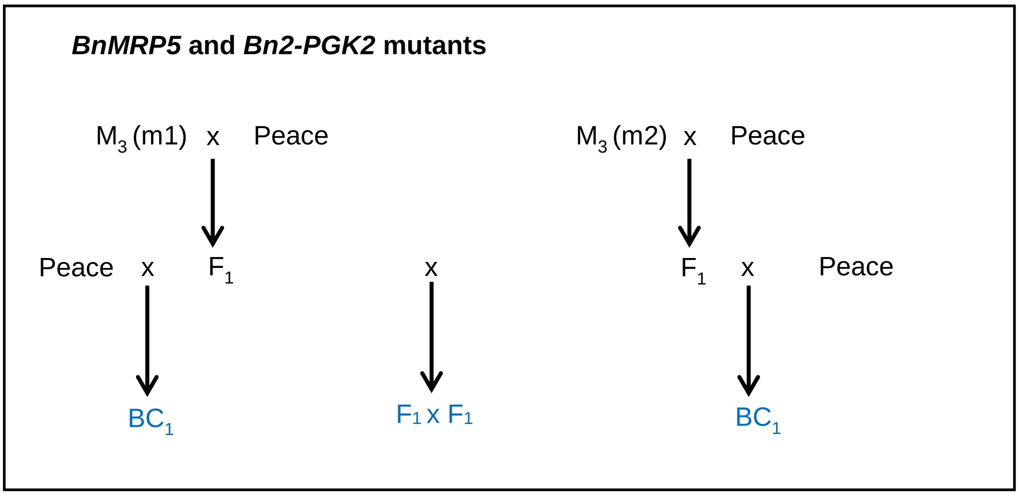

Supplementary Fig. 1: Crossing schemes for producing single and double mutants and for backcrossing with Peace. After each crossing, heterozygous mutants were selected with molecular markers. Populations used for genomic background genotyping are marked in blue. 'm1' and 'm2' indicate the two different M_3 mutant alleles selected for crossing.

References

1 Karunarathna, N. L., Wang, H. Y., Harloff, H. J., Jiang, L. X. & Jung, C. Elevating seed oil content in a polyploid crop by induced mutations in *SEED FATTY ACID REDUCER* genes. *Plant Biotechnol J*, doi:10.1111/pbi.13381 (2020).

2 Sashidhar, N., Harloff, H.-J. & Jung, C. Identification of phytic acid mutants in oilseed rape (*Brassica napus*) by large scale screening of mutant populations through amplicon sequencing. *New Phytologist* **n/a**, doi:10.1111/nph.16281 (2019).

3 Sashidhar, N., Harloff, H. J. & Jung, C. Knockout of *MULTI-DRUG RESISTANT PROTEIN 5* Genes Lead to Low Phytic Acid Contents in Oilseed Rape. *Front Plant Sci* **11**, doi:10.3389/fpls.2020.00603 (2020).

4 Liu, J. *et al.* An improved allele-specific PCR primer design method for SNP marker analysis and its application. *Plant Methods* **8**, 34, doi:10.1186/1746-4811-8-341746-4811-8-34 [pii] (2012).
